# Pharmacokinetic–pharmacodynamic analysis of cefmetazole against extended-spectrum β-lactamase-producing Enterobacteriaceae in dogs using Monte Carlo Simulation

**DOI:** 10.3389/fvets.2023.1270137

**Published:** 2023-09-28

**Authors:** Mizuki Kusumoto, Tomoki Motegi, Haruna Uno, Mizuki Yokono, Kazuki Harada

**Affiliations:** ^1^Laboratory of Veterinary Internal Medicine, Tottori University, Tottori, Japan; ^2^Joint Graduate School of Veterinary Sciences, Tottori University, Tottori, Japan; ^3^Department of Veterinary Clinical Pathobiology, Graduate School of Agricultural and Life Sciences, The University of Tokyo, Bunkyo, Japan; ^4^Technical Department, Tottori University, Tottori, Japan

**Keywords:** cefmetazole, extended-spectrum β-lactamase, Enterobacteriaceae, Monte Carlo Simulation, dog, pharmacokinetics, pharmacodynamics

## Abstract

**Introduction:**

The spread of extended-spectrum β-lactamase-producing Enterobacteriaceae (ESBL-E) is a serious concern in companion animal medicine owing to their ability to develop multidrug resistance. Cefmetazole (CMZ) is a candidate drug for treating ESBL-E infections; however, its regimen in dogs has not been established. In this study, we investigated the pharmacokinetic (PK) indices of CMZ in dogs and performed PK–pharmacodynamic (PD) analyses using Monte Carlo Simulation (MCS).

**Methods:**

In total, six healthy dogs received an intravenous bolus dose of CMZ (40 mg/kg body weight). Serum CMZ concentrations were evaluated using liquid chromatography–mass spectrometry, and PK indices were determined based on non-compartmental analysis. The PK–PD cut-off (COPD) values were calculated as the highest minimum inhibitory concentration (MIC) that achieved ≥90% probability of target attainment for a target value of unbounded drug concentration exceeding 40% of the dosing interval. The cumulative fraction of response (CFR) was calculated based on the MIC distribution of wild-type ESBL-E from companion animals.

**Results:**

The area under the concentration–time curve and elimination half-time were 103.36 ± 7.49 mg·h/L and 0.84 ± 0.07 h, respectively. MCS analysis revealed that COPD values for regimens of 40 mg/kg q12, q8h, and q6h were ≤ 0.5, ≤2, and ≤ 4 μg/mL, respectively. A regimen of 40 mg/kg q6h was estimated to achieve a CFR of 80–90% for *Escherichia coli* and *Klebsiella pneumoniae*. By contrast, all regimens exhibited a CFR of ≤70% for *Proteus mirabilis* and *Enterobacter cloacae.*

**Discussion:**

We conclude that CMZ at 40 mg/kg q6h could be a viable treatment regimen for dogs infected with ESBL-producing *Escherichia coli* and *Klebsiella pneumoniae*.

## 1. Introduction

The spread of extended-spectrum β-lactamase (ESBL)-producing Enterobacteriaceae (ESBL-E) has emerged as a serious concern in companion-animal and human medicine (1–3). Plasmids encoding ESBLs often carry resistance genes for different classes of antimicrobials; therefore, ESBL-E have developed multidrug resistance with high resistance against β-lactams and other classes of antimicrobials, thus limiting the number of effective antimicrobial agents available for treatment (1). Carbapenems are recommended as the first-line drugs for treating ESBL-E infections in humans but not in animals owing to the risk of developing carbapenemase-producing Enterobacteriaceae (CPE), which is a more serious threat than ESBL-E (4, 5). Therefore, the use of carbapenems is discouraged in veterinary medicine due to the potential risk of zoonotic transmission from companion animals to humans through close contact (6). Thus, exploring alternatives to carbapenems is crucial in veterinary medicine.

Cefmetazole (CMZ) belongs to the group of cephamycins and exhibits high stability against β-lactamases due to the presence of the 7α methoxy constituent (7, 8). A previous study in humans has reported that compared with carbapenems, CMZ exhibits non-inferior clinical efficacy for treating ESBL-E infections (9). Furthermore, we previously investigated the pharmacodynamics (PD) of CMZ and found that the drug exhibits high *in vitro* efficacy against ESBL-E isolates from companion animals (10, 11), as well as some essential oils (12). These findings suggest that CMZ can be used as a carbapenem-sparing drug in companion-animal medicine. However, CMZ regimens for treating ESBL-E infections in dogs have not been established because the pharmacokinetics (PK) of CMZ in dogs is not known.

Recently, PK–PD analysis using Monte Carlo Simulation (MCS) has been used to study appropriate dosing regimens of antimicrobial drugs (13, 14). MCS can establish large virtual populations via the randomization of PK and PD indices and thereby estimate the probability of achieving antimicrobial efficacy (probability of target attainment, PTA) by dosage regimen (14). In this study, we calculated the PK indices of CMZ in dogs after intravenous administration study. To the best of our knowledge, this is the first study to perform PK–PD analysis using MCS to determine the PK–PD cut-off value (COPD) and propose dosing regimens of CMZ which can be clinically effective for ESBL-E infections in dogs.

## 2. Materials and methods

### 2.1. Animals

In total, six healthy beagles (4 males, 2 females, aged 5.3 ± 2.0 years and weighing 12.5 ± 1.8 kg, SHIMIZU Laboratory Supplies Co., Ltd., Kyoto, Japan) were used in this study. The dogs were clinically healthy based on their condition, physical examination, complete blood counts, and blood biochemical tests and did not receive any drugs in the 6 months before the study. They were fed the same commercial food (Aiken Genki, Unicharm Corporation, Tokyo, Japan) and were individually housed in separate cages in the same room at the experiment animal facility. This study was conducted under an ethics committee-approved protocol in accordance with the Tottori University Animal Use Committee (approval number No. 19-T-17).

### 2.2. Drug administration and serum sampling

The day before drug administration, a central venous catheter (Covidien Japan, Inc., Tokyo, Japan) was placed in the jugular vein of the dog under general anesthesia. Anesthesia was induced by intravenously administering propofol (4 mg/kg body weight, Propoflo, DS Pharma Animal Health Co., Ltd., Osaka, Japan), and subsequently intubated with a cuffed endotracheal tube. The vaporizer was adjusted to deliver 2% isoflurane (ISOFLURANE Inhalation Solution, Mylan EPD G.K., Tokyo, Japan) at an oxygen flow rate of 2 L/min. CMZ sodium (Nichi-Iko Pharmaceutical Co., Ltd., Tokyo, Japan) was dissolved in water for a bolus injection (Nissin Pharmaceutical Co., Ltd., Yamagata) at a final concentration of 100 mg/mL, which was injected in the radial cutaneous vein at 40 mg/kg body weight. For CMZ quantification, venous blood samples (2 mL each) were collected from each participant via a central venous catheter at predetermined time points (0, 5, 10, 15, 30, 45, 60, 90, 120, 150, 180, 240, 360, 480, 600, and 720 min). Serum samples were obtained after coagulation and then frozen at −80°C until quantification.

### 2.3. Sample preparation

CMZ was extracted from each serum sample using solid-phase extraction (SPE) according to a previously described protocol (15). Ethylparaben (Sigma-Aldrich Japan, Tokyo, Japan) was used as an internal standard (IS), which was dissolved in methanol to 10 μg/mL, because the compound is stable and shows a retention time (6.36 min) close to that of CMZ (3.6 min). The prepared sample was stored frozen at −80°C until analysis.

### 2.4. Determination of antimicrobial concentrations

The serum concentration of CMZ was determined according to a previously described protocol with slight modifications (15). High-performance liquid chromatography (HPLC) separations were performed with a 10A HPLC system (Shimadzu, Kyoto, Japan) under 50:50 isocratic conditions using the following two solutions: 0.1% (v/v) formic acid in 10 mM ammonium formate water as mobile phase A and 0.1% (v/v) formic acid-acetonitrile as mobile phase B at a flow rate of 0.2 mL/min. A 20 μL sample of serum was injected and target molecules were separated using TSKgel Reversed Phase Chromatography (TSKgel ODS-100Z 3 μm, 2.0 mm i.d. x 150 mm, TOSOH, Tokyo, Japan), with the temperature controlled at 40°C. Mass spectra (MS) were measured using an Exactive mass spectrometer (Thermo Fisher Scientific, Waltham, MA, United States) under ESI with a tube lens voltage of 80 V and a skimmer voltage of 30 V. CMZ was detected by [M + H]^+^ precursor ion m/z = 472.1, and IS was detected by [M-H]^−^ precursor ion m/z = 165.1. The area under the peak was determined using commercial analytical software (Xcalibur QualBrowser, Thermo Fisher Scientific Inc., MA, United States). The concentration of CMZ in each sample was calculated based on the calibration curve (0.1, 1, 5, 10, 50, 100, and 200 μg/mL) constructed by mixing canine serum with the known concentrations of the drug. The reliability of the analytical method was based on the guidelines of the Ministry of Health, Labor, and Welfare (MHLW) (16).

### 2.5. Monte Carlo Simulation

MCS was performed to calculate PTA based on the PK–PD parameters of CMZ when administered at 40 mg/kg body weight at q12h, q8h, and q6h, using commercial software (Oracle Crystal Ball version 11.1.2.4.850, Kozo Keikaku Engineering Inc., Tokyo, Japan). The PK parameters in the non-compartment model (17) were calculated based on the serum CMZ concentrations in the six dogs by computing 10,000 bootstrap replicates using the PK package (ver. 4.0.3) of R software (ver. 4.2.1) (18). Assuming that PK parameters were distributed lognormally, 10,000 virtual patients were generated for each dosage regimen to build the drug serum concentration-time profile. The percentage of time for which the unbound drug concentrations remained above the minimum inhibitory concentration (MIC) (fTAM) was estimated as the PK–PD index to determine the optimal dosage regimen (19, 20). In this study, a serum protein-binding rate of 26% was applied according to a previous study (21). The PTA for each dosing regimen was calculated at each MIC to determine the percentage of subjects achieving the PK–PD index that could achieve bacteriostatic activity (i.e., fTAM ≥40%) (19, 20). COPD was calculated as the highest MIC that achieved the target of PTA ≥ 90% (20). The cumulative fraction of response (CFR) was calculated as the proportion of %PTA of each MIC based on the wild-type MIC distribution (22), of which CMZ in ESBL-E (*Escherichia coli*, *Klebsiella pneumoniae*, *Proteus mirabilis*, and *Enterobacter cloacae*) isolates from companion animals were determined in the previous studies (10, 11).

## 3. Results

None of the dogs exhibited clinical signs of adverse reactions or abnormal blood test results throughout the experiment.

**Figure 1:**
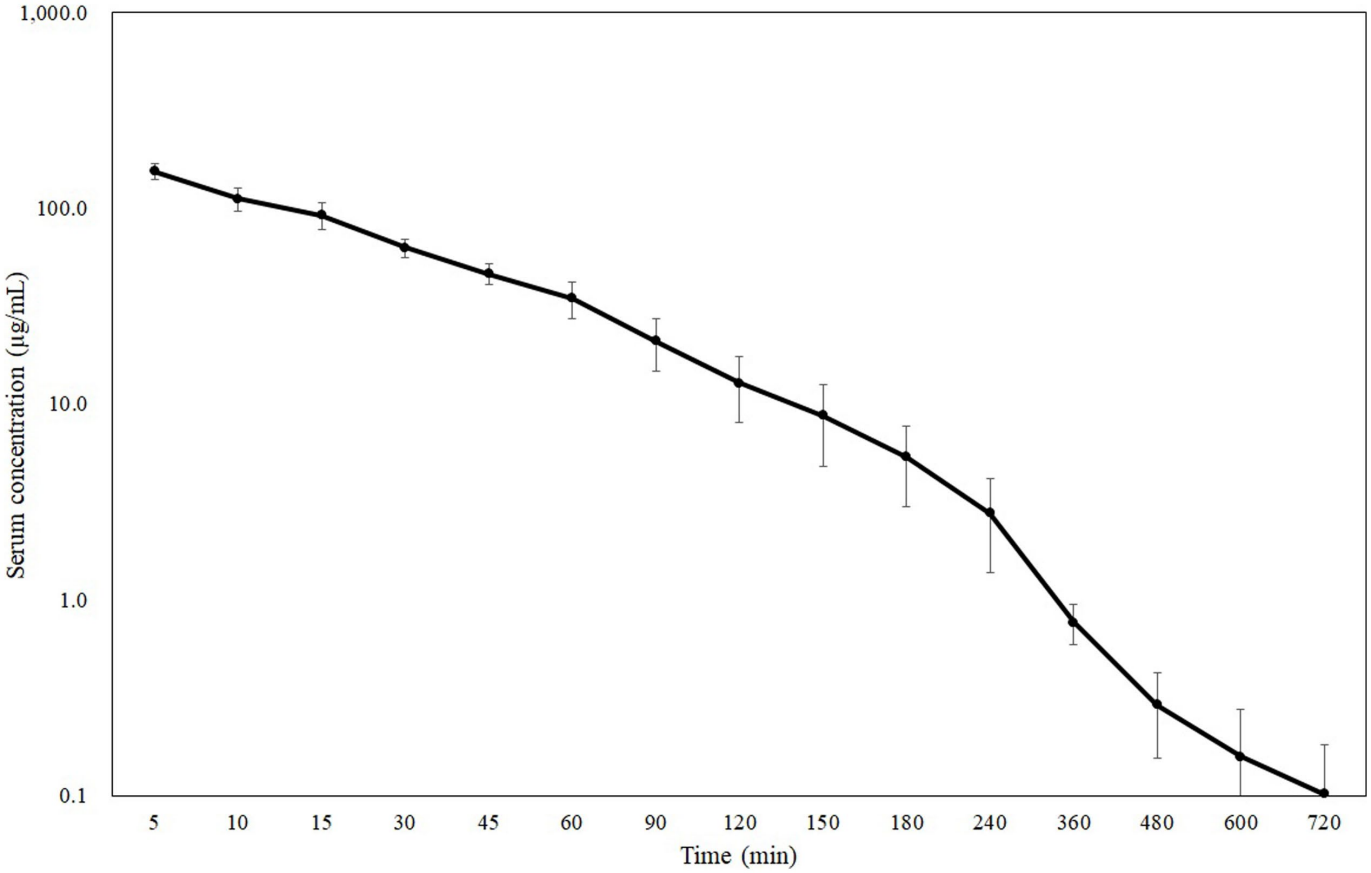
Semilogarithmic plot of serum CMZ concentration in dogs administered a dose of 40 mg/kg body weight (Mean ± SD, *n* = 6).

The coefficient of variation (CV) of CMZ, IS, and CMS peak area value corrected by IS (CMZ/IS) was 4.01, 12.1, and 9.50%, respectively, all of which fulfilled the criteria (< 15%) according to the MHLW guideline (16). The blood concentration-time curves and PK parameters in dogs after intravenous bolus administration of CMZ at 40 mg/kg are presented in Figure 1 and Table 1, respectively. Serum CMZ concentration at 5 min was 154.32 ± 14.01 μg/mL, which decreased gradually.

**Table 1:** Pharmacokinetic parameters determined after intravenous administration of CMZ at the dose of 40 mg/kg body weight in dogs.

Parameters (unit)	Values (SD)
AUC (mg·h/L)	103.36 (7.49)
MRT (h)	1.21 (0.11)
T1/2 (h)	0.84 (0.07)
CL (L/h)	4.93 (0.36)
Vd (L)	5.97 (0.55)

Values are the mean (SD)from six dogs after intravenous administration.

AUC, area under the concentration-time curve; MRT, mean residence time; T1/2, elimination half-life; CL, total body clearance; Vd, the volume of distribution.

We previously determined MICs of CMZ in a total of 308 ESBL-producing isolates of *Escherichia coli* (*n* = 90), *Klebsiella pneumoniae* (*n* = 120), *Proteus mirabilis* (*n* = 29), and *Enterobacter cloacae* (*n* = 69), using the agar dilution method according to the Clinical and Laboratory Standards Institute guideline (10, 11). These investigations showed that the MIC_50_ and MIC_90_ of CMZ were 1 and 8 μg/mL for *Escherichia coli*, 2 and 32 μg/mL for *Klebsiella pneumoniae*, 4 and 32 μg/mL for *Proteus mirabilis*, and 256 and > 256 μg/mL for *Enterobacter cloacae*. In this study, we observed the mean blood concentrations decreased below the MIC_90_ for *Klebsiella pneumoniae* and *Proteus mirabilis* at 1 h and below that for *Escherichia coli* at 3 h. Furthermore, the serum concentration decreased below the MIC_50_ for *Proteus mirabilis* at 4 h and below that for *Escherichia coli* and *Klebsiella pneumoniae* at 6 h. In contrast, the mean blood concentration at 5 min did not exceed the MIC_50_ and MIC_90_ for *Enterobacter cloacae*.

**Figure 2:**
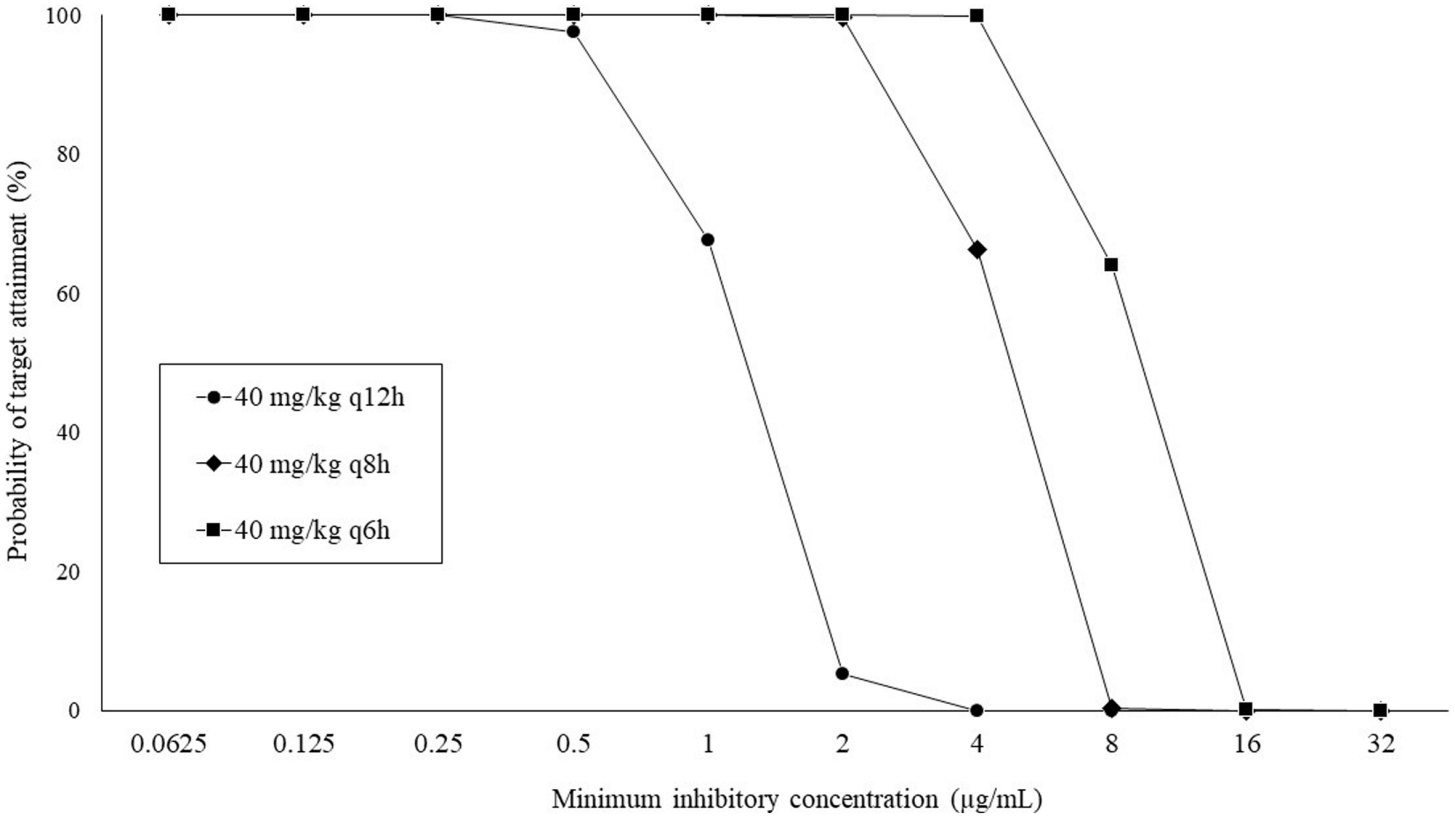
Probability of target attainment (%) at each minimum inhibitory concentration following intravenous administration of CMZ.

The PTA results at each MIC with regimens of 40 mg/kg q12h, q8h, and q6h are shown in Figure 2. All regimens achieved a PTA of ≥90%, with a MIC of ≤0.5 μg/mL but not with a MIC of ≥8 μg/mL. Based on the calculated PTA, the COPD values for q12, q8h, and q6h were ≤ 0.5, ≤2, and ≤ 4 μg/mL, respectively.

Table 2 shows the CFR results that were calculated based on the wild-type MIC distribution of ESBL-E. Overall, higher CFRs were observed for *Escherichia coli* and *Klebsiella pneumoniae* than for *Proteus mirabilis*, and extremely low CFRs (<10%) were confirmed for *Enterobacter cloacae*, regardless of the regimen. When using fTAM ≥40% as the target of the PK–PD index, none of the regimens achieved a CFR ≥90% in canines infected with all bacterial species. Regimens of 40 mg/kg q6h and q8h were estimated to achieve CFR of 80–90% and 70–80%, respectively, for patients infected with *Escherichia coli* and *Klebsiella pneumoniae*. In contrast, all regimens were estimated to have a CFR of ≤70% for patients infected with *Proteus mirabilis* and *Enterobacter cloacae*.

**Table 2:** Cumulative fraction of response following regimens of 40 m/kg CMZ in dogs against wild-type MIC distribution of ESBL-producing Enterobacteriaceae.

Regimens	Cumulative fraction of response (%)			
	*Es. coli*	*K. pneumoniae*	*P. mirabilis*	*En. cloacae*
q12h	31.91	26.86	1.76	2.38
q8h	78.12	72.91	48.29	6.57
q6h	87.27	82.29	63.73	7.24

## 4. Discussion

There is a growing demand for alternative carbapenem-based antibiotics to appropriately treat ESBL-E infections without spreading CPE (5). Unfortunately, these antibiotics have not been thoroughly investigated in veterinary medicine. CMZ is one of the candidate drugs to spare carbapenems because the drug is highly stable against ESBLs (5). However, PK-PD properties of the drug, which are essential to consider optimal dosing regimens (23, 24), are still unknown. To resolve this issue, we investigated the PK indices of CMZ in dogs and performed PK–PD analyzes using MCS. Furthermore, we estimated the COPD values of CMZ when administered with expected regimens using MCS and calculated the CFR based on the MIC distribution of wild-type ESBL-E.

We investigated the PK parameters of CMZ in dogs based on the results of blood concentration transition after bolus administration. Borin et al. (25) determined the corresponding PK data in young adult humans, ranging in age from 21 to 40 years, when intravenously administered, as follows: the mean residence time (MRT) was 1.78 h, the elimination half-life (T1/2) was 1.34 h, total body clearance (CL) was 0.10 L/h/kg, and the volume of distribution (Vd) was 0.17 L/kg. Our study indicates the shorter MRT and T1/2, higher CL, and larger Vd in dogs administered with CMZ, compared with the PK data in humans. These results may be explained by the fact that the protein-binding rate of CMZ is significantly lower in dogs than in humans (66%) (21), which can result in wider tissue distribution and faster renal excretion. These PK traits of CMZ in dogs may negatively affect its clinical efficacy because its bactericidal activity is time-dependent (26).

Breakpoints of antimicrobial susceptibility are essential for appropriate antimicrobial treatment. The Clinical Laboratory Standards Institute (27) provided the human-specific breakpoint of the CMZ but not the canine-specific breakpoint. Therefore, we studied the canine-specific COPD values of CMZ for practical application in dogs with bacterial infections. Our finding revealed that COPD values can be increased by shortening dose interval; however, COPD values of CMZ based on all dosage regimens were lower than the CLSI susceptibility breakpoint for Enterobacteriaceae (≤16 μg/mL), probably due to the higher clearance of the drug in dogs. Therefore, the application of the CLSI breakpoint may overestimate CMZ susceptibility in canine pathogens, resulting in treatment failure.

The present COPD values with all regimens were also lower than the previously reported MIC_90_ values of ESBL-producing *Escherichia coli* (8 μg/mL), *Klebsiella pneumoniae* (32 μg/mL), *Proteus mirabilis* (32 μg/mL), and *Enterobacter cloacae* (>256 μg/mL) (10, 11). Such lower COPD values were supported by other findings in this study that CMZ exhibited a relatively short time during which the blood concentration remained above the MIC_90_ of these bacteria. When administered with the regimens of q6h and q8h, the COPD induced by CMZ exceeded the MIC_50_ values of ESBL-producing *Escherichia coli* (1 μg/mL) and *Klebsiella pneumoniae* (2 μg/mL), but not *Proteus mirabilis* (4 μg/mL) and *Enterobacter cloacae* (256 μg/mL). Furthermore, we calculated the CFR based on the wild-type MIC distribution of ESBL-E and thereby clarified that the q6h regimens exhibited more than 80% CFRs for ESBL-producing *Escherichia coli* and *Klebsiella pneumoniae.* Generally, regimens with ≥90% CFR are optimal, whereas those with 80–90% CFR are associated with moderate probabilities of success (28). Therefore, at least the regimens of CMZ 40 mg/kg q6h may have moderate clinical efficacy in canine patients infected with ESBL-producing *Escherichia coli* and *Klebsiella pneumoniae*. However, all regimens in this study exhibited lower CFRs against ESBL-producing *Proteus mirabilis* and *Enterobacter cloacae* and thus may have less efficacy for these bacteria. Such differences in the putative clinical efficacy of CMZ between bacteria should be considered when administering CMZ in dogs.

Unfortunately, the optimal dose of CMZ in dogs has not yet been determined. In previous reports (29–31), CMZ was administered to canine patients at 20–25 mg/kg body weight per dose. However, these usages were intended for the perioperative prevention of infection but not for the treatment of bacterial infection. Our preliminary study showed that COPD and CFR at a dose of 20 mg/kg were extremely low (data not shown); thus, this dosage is unlikely to be suitable for treating dogs with ESBL-E infections. Therefore, we used 40 mg/kg per dose in this study, referencing the human dosage (i.e., a maximum of 37.5 mg/kg four times per day). This dose is considered acceptable from a safety perspective because CMZ has a wide margin of safety (32) and extremely high level of no observed effect in dogs (i.e., 1,200 mg/kg) (33).

We applied a fTAM of ≥40% as the PK–PD target values (19, 20), but McKinnon et al. (34) reported that a fTAM of 100% is recommended as the target value of cefepime and ceftazidime for serious infection. Furthermore, in recent years, Takemura et al. (35) demonstrated that a fTAM of >57.6% was preferable as the target value of CMZ for static effects on ESBL-producing *Es. coli* infections in a murine infection model. These studies emphasize the need to consider higher PK–PD target values for CMZ in dogs. More than half of dog-origin ESBL-E strains have been isolated from urinary tract infections (10, 11). In addition, the peak drug concentration is approximately 20 times higher in the urine than in the blood of dogs after administration of CMZ (36), implying that CMZ may have high efficacy against urinary tract infection in dogs with ESBL-E, even if the blood concentration during repeated administration cannot achieve the target value. Further clinical studies are required to confirm this hypothesis.

Our study has several limitations. We only used a small number of dogs to calculate the PK–PD parameters due to animal welfare concerns. However, we increased the reliability of these parameters by using bootstrap replicates. Furthermore, PK parameters were determined in healthy beagle dogs, which may differ from those in patients with renal dysfunction, as previously reported in humans (37). Monaghan et al. (38) demonstrated that similar to that of CMZ, the blood concentration of ampicillin, an antibiotic that is mainly excreted renally, can increase in dogs with renal diseases. Such increased antibiotic concentrations may result in increased efficacy of the drug. Lastly, MIC-based PK-PD analysis was performed in this study; therefore, we did not consider other PD parameters, such as multiple PD parameters and kill rate (39).

To conclude, we estimated the COPD of CMZ when administered with regimens of 40 mg/kg q6h, q8h, and q12h using MCS and calculated the CFR based on the MIC distribution of wild-type ESBL-E. Our results indicated that a CMZ breakpoint lower than the CLSI for humans is preferable for dogs. Furthermore, a CMZ regimen of 40 mg/kg q6h could be a treatment option for dogs infected with ESBL-producing *Escherichia coli* and *Klebsiella pneumoniae*. We believe that these data provide a basis for the use of CMZ in dogs with ESBL-E infections.

## Data availability statement

The raw data supporting the conclusions of this article will be made available by the authors, without undue reservation.

## Ethics statement

The animal study was approved by Tottori University Animal Use Committee. The study was conducted in accordance with the local legislation and institutional requirements.

## Author contributions

MK: Data curation, Investigation, Methodology, Writing – original draft. TM: Conceptualization, Formal analysis, Software, Visualization, Writing – review & editing. HU: Data curation, Investigation, Methodology, Writing – review & editing. MY: Data curation, Investigation, Methodology, Writing – review & editing. KH: Conceptualization, Data curation, Formal analysis, Funding acquisition, Investigation, Methodology, Software, Visualization, Writing – original draft, Writing – review & editing.
